# Disrupted topological organization of the motor execution network in Wilson's disease

**DOI:** 10.3389/fneur.2022.1029669

**Published:** 2022-11-21

**Authors:** Long Zhu, Hongxi Yin, Yanxin Wang, Wenming Yang, Ting Dong, Lei Xu, Zhifeng Hou, Qiao Shi, Qi Shen, Zicheng Lin, Haixia Zhao, Yaqin Xu, Yanyan Chen, Jingjing Wu, Zheng Yu, Man Wen, Jiaying Huang

**Affiliations:** Department of Encephalopathy, The First Affiliated Hospital of Anhui University of Traditional Chinese Medicine, Hefei, China

**Keywords:** Wilson's disease, resting-state functional magnetic resonance imaging, motor execution network, small-world attributes, global topological organization, nodal topological organization

## Abstract

**Objective:**

There are a number of symptoms associated with Wilson's disease (WD), including motor function damage. The neuropathological mechanisms underlying motor impairments in WD are, however, little understood. In this study, we explored changes in the motor execution network topology in WD.

**Methods:**

We conducted resting-state functional magnetic resonance imaging (fMRI) on 38 right-handed individuals, including 23 WD patients and 15 healthy controls of the same age. Based on graph theory, a motor execution network was constructed and analyzed. In this study, global, nodal, and edge topological properties of motor execution networks were compared.

**Results:**

The global topological organization of the motor execution network in the two groups did not differ significantly across groups. In the cerebellum, WD patients had a higher nodal degree. At the edge level, a cerebello-thalamo-striato-cortical circuit with altered functional connectivity strength in WD patients was observed. Specifically, the strength of the functional connections between the cerebellum and thalamus increased, whereas the cortical-thalamic, cortical-striatum and cortical-cerebellar connections exhibited a decrease in the strength of the functional connection.

**Conclusion:**

There is a disruption of the topology of the motor execution network in WD patients, which may be the potential basis for WD motor dysfunction and may provide important insights into neurobiological research related to WD motor dysfunction.

## Introduction

Hepatolenticular degeneration (HLD), also known as Wilson's disease (WD), is a rare autosomal recessive metabolic disease in adolescents, which is mainly characterized by liver damage and movement disorders (1). The liver and brain are the main organs that are damaged in WD patients, and the most commonly affected parts of the brain are the basal ganglia, where the striatum, amygdala and claustrum are located. Although the basal ganglia are most commonly affected, cerebral atrophy is also a common finding. For example, SmolinskiLukasz et al. (2) found that the severity of cerebral atrophy is closely related to the neurological impairment of WD patients by measuring the brain volume of WD patients. Later, SmolinskiLukasz et al. (3) conducted a long-term longitudinal study on brain atrophy in WD patients, and found that the incidence of brain atrophy in neurological WD patients was significantly increased, which was related to the progression of neurological impairment. Almost all patients with neurological WD show brain MRI changes (4). On MRI images of WD patients, symmetrical T1 hypointensity and T2 hyperintensity were observed in the lenticular nucleus. Depending on the different nuclei involved, the lesions show signs like “woodpecker”, “figure eight”, “double figure eight” and “butterfly with wings”, and MRI enhanced scan shows that the lesions are not enhanced (5). Abnormalities in corpus callosum signals suggest a wider range of brain damage and neurological dysfunction (6). In addition, WD patients may have different degrees of cerebral atrophy such as narrowing of gyri and deepening of sulci (7–9). However, brain atrophy is not limited to the cortical region. Volume measurement of corresponding parts on MRI images of WD patients showed that the volume of caudate nucleus, globus pallidus and thalamus decreased (10). Approximately half of WD patients have different degrees of neurological symptoms, the common manifestations of which are dystonia, tremor, limb stiffness, bradykinesia and other rare neurological symptoms (11). Dystonia can show blepharospasm (eye muscles), torticollis (neck muscles), writing spasm (hand muscles), and exaggerated facial expressions (facial muscles) due to the different muscle groups involved. When the muscles around the vocal cords are involved, dyspnea and dysarthria can occur. Tremors can occur in both stationary and motor state, and the common ones are essential tremors and postural tremors. Some WD patients show parkinsonian symptoms such as limb stiffness, bradykinesia and slow walking, which are easy to be misdiagnosed. Ataxia and other neurological symptoms are rare (5). Neurological manifestations are equally varied but are generally dominated by movement disorders (12). Converging evidence indicates a definite association between Wilson's disease and motor impairments. A study of non-motor symptoms in WD demonstrated that movement disorders are the core neurological features of the disease (13). Additionally, a molecular genetics study of WD found that WD was caused by mutations in a large number of different genes, which made the molecular diagnosis complicated to achieve. The genetic map of dopa-responsive dystonia has been mapped, however, the genetic basis of many movement disorders, such as essential tremor and restless legs syndrome, remains unclear (14). A study on eye movement performance in WD found that patients with WD had ocular saccade impairments, including latencies, hypsometry and increased error rates in antisaccades (15). Another large sample study on eye movements in WD disease found that WD was associated with impaired voluntary control of saccades and disturbance of smooth chase eye movements, while reflex saccades seemed to be preserved (16). Medalia et al. found that the independent sequelae of copper induced central nervous system (CNS) injury were motor, memory and psychiatric symptoms (17). A multimodal magnetic resonance study found that the asymmetry of fiber projection may be the main cause of motor asymmetry in WD patients (18). Although these findings exist, the neuropathological mechanisms underlying WD motor disorders are still poorly understood.

Human brains are increasingly recognized to be intricate networks of highly connected neurons (19). Graph theory has been introduced as a powerful method for the study of the complexity of brain networks, with nodes representing anatomically defined brain areas and edges representing functional or structural connections between pairs of nodes (20–23). The application of graph-theoretic methods facilitates the systematic description of topological properties of brain networks from global, node, and edge perspectives, which cannot be achieved by using independent component analysis or seed-based functional connectivity analysis. *via* of these methods, complex brain networks have been shown to exhibit high global integration and high local properties, thereby supporting high efficiency at lower wiring costs (24–30). In our previous study, we applied graph theory to unravel abnormalities in brain networks in patients with WD previously. For example, in the study of changes in the global topological properties of functional and structural networks in WD patients, we found that both functional and structural networks have typical small-world properties in both groups. What's more, compared with healthy controls, WD patients exhibited disruptions of structural networks, which was characterized by an increased clustering coefficient and characteristic path length and decreased global and local efficiency. In past graph theoretical studies of WD patients, however, whole-brain networks have been studied rather than subnetworks containing specific functions, such as motor control. The latter aspect may contribute to our understanding of the underlying neural mechanisms of WD-related symptoms, such as dyskinesia symptoms.

Our study focused on the motor execution network since WD and motor impairments are closely related, and we hypothesized that motor execution networks in WD patients would be abnormal. We applied graph theory to resting-state fMRI data, aiming to detect group differences in the topological properties of exercise executive networks in WD patients and healthy controls.

## Methods

### Participants

The present study enrolled 38 right-handed individuals, including 23 patients with WD and 15 healthy controls. To ensure that WD patients could receive MRI scans and to reduce the artifacts generated by head movements as much as possible, 23 WD patients with motor impairments with decreased head vibration were selected for the experiment. WD patients with a mean age of 22.3 years (range: 15–36 years) were recruited from the inpatient department at The First Affiliated Hospital of Anhui University of Chinese Medicine. Only hnospitalized patiets were selected because they were extreme cases, who may have more typical and defined brain dysfunction. The participants were recruited from the hospital and schools through advertising and had an average age of 24.67 years (range: 15–36 years). The study was conducted in accordance with the Declaration of Helsinki and was approved by the Medical Research Ethics Committee of First Affiliated Hospital of Anhui University of Chinese Medicine. Every subject was informed of the study and given a written consent.

The diagnoses of WD were determined according to The Guidelines for the Diagnosis and Treatment of Hepatolenticular Degeneration in China 2021 (31). The inclusion criteria for WD patients were as follows: ① age of 15–36-years-old; ② right-handed; ③ patients diagnosed with WD for the first time or those patients diagnosed with WD in the past but who had stopped drug therapy for more than 1 year; and ④ the UWDRS-IB score (32) suggests motor dysfunction. Healthy controls were included with the following criteria: ① age of 15–36-years-old; ② right-handed; and ③ no obvious physical or mental disorders. The following exclusion criteria were used for all of the participants: a history of head trauma with impaired consciousness lasting more than 5 min, a history of drug or alcohol abuse, pregnancy and any physical illness diagnosed *via* interviews and medical records review. Additional exclusion criteria for all of the participants included a state of anxiety and depression, which were diagnosed by using the 24-item Hamilton Rating Scale for Depression (HAMD) (33) and the 14-item Hamilton Rating Scale for Anxiety (HAMA) (34). The HAMD is a score for the degree of depression of the subjects. When the score was >8 points (which indicates that there may be depression), this patient was excluded. The HAMA is a score for the degree of anxiety of the subjects. When the score was more than 7 points (which indicates that there may be anxiety), this patient was excluded.

### MRI data acquisition

MRI data were acquired by using a 3.0-Tesla MR system (Discovery MR750, General Electric, Milwaukee, WI, USA). We used tight, comfortable foam padding to reduce subject head movement, and earplugs were used to reduce noise from the scanner. 3D T1-weighted images were acquired by using a brain volume (BRAVO) sequence with the following parameters: repetition time (TR) = 8.16 ms; echo time (TE) = 3.18 ms; inversion time (TI) = 450 ms; flip angle (FA) = 12°; field of view (FOV) = 256 mm × 256 mm; matrix = 256 × 256; slice thickness = 1 mm, no gap; 184 sagittal slices; and acquisition time = 357 s. BOLD images were acquired by using a GRE-SS-EPI sequence with the following parameters: TR/TE = 2,000/35 ms; FOV = 220 mm × 220 mm; matrix = 64 × 64; FA = 90°; slice thickness = 3 mm; gap = 0.5 mm; 36 interleaved transverse slices; 185 volumes; and acquisition time = 370 s. We asked all subjects to close their eyes during the MRI scan, move as little as possible, not think about anything in particular, but not fall asleep. To ensure that only images without visible artifacts were included in the analysis, all MR images were visually inspected.

### FMRI data preprocessing

Resting-state BOLD data preprocessing step used Statistical Parametric Mapping 12 (SPM12, http://www.fil.ion.ucl.ac.uk/spm). The following steps of the analysis were performed. ① Slice timing. The first 10 time points were removed to allow the signal to equilibrate and to acclimate the participants to the scanning noise. The remaining time points were corrected for the acquisition time delay between layers. ② Realignment. After slice timing, the motion between the time points was corrected by realignment. During realignment, three translational and three rotational motion parameters were computed. Head movements of all participants' BOLD signal data were within thresholds (i.e., translational or rotational parameters <2 mm or 2°). ③ Normalization. In the normalization step, the personal structural image was first registered to the average functional image. Subsequently, the transformed structural image was segmented by using the DARTEL technique, and the standard Montreal Neurological Institute (MNI) space was obtained (35). Finally, by using the above two deformation parameters estimated in the first step, each filtered feature image space was normalized to MNI space and resampled to 3 × 3 × 3 mm cubic voxels. ④ Detrending. ⑤ Filtering. The datasets were then bandpass filtered in a frequency range of 0.01–0.08 Hz. ⑥ Regression. Several nuisance covariates (the estimated motion parameters based on the Friston-24 model, the white matter signal and the cerebrospinal fluid signal) were regressed out from the data. We also calculated the framewise displacement (FD), which indices the volume-to-volume changes in the head position. There was no significant difference in the mean FD (Wilcoxon rank-sum test of two independent samples, *Z* = −0.923, 0.356) between the WD (0.093 ± 0.043) group and the control (0.104 ± 0.048) group. It has been reported that signal spikes caused by head movement can significantly affect the final resting-state fMRI results, even after regression of linear head movement parameters (36). Consequently, we further regressed the spikes when the FD at a particular time point exceeded 0.5.

### Construction of motor execution network

**Figure 1:**
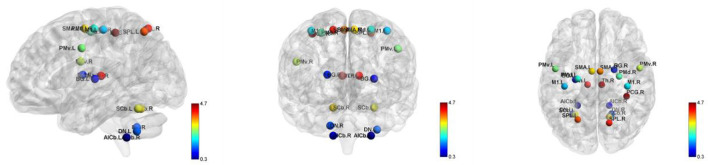
Locations of the nodes belonging to the motor execution network. AICb, anterior inferior cerebellum; BG, basal ganglia; DN, dentate nucleus; L, left; M1, primary motor cortex; PCG,postcentral gyrus; PMd, dorsolateral premotor cortex; PMv, ventrolateral premotor cortex; R, right; SCb,superior cerebellum; SMA, supplementary motor area; SPL, superior parietal lobule; Th, thalamus.

Based on a previous study, we selected the motor execution network regions of interest (ROIs) for node definition (37). The ROIs included 21 regions, such as the bilateral anterior inferior cerebellum (AICb; MNI coordinate: left−22/-45/-49, right 16/-45/-49), basal ganglia (BG; left−25/-14/8, right 22/-2/12), dentate nucleus (DN; left−28/-55/-43, right 19/-55/-39), supplementary motor area (SMA; left−5/-4/57, right 5/-4/57), superior parietal lobule (SPL; left−22/-62/54, right 16/-66/57), primary motor cortex (M1; left−38/-22/56, right 38/-22/56), dorsolateral premotor cortex (PMd; left−22/-13/57, right 28/-10/54), ventrolateral premotor cortex (PMv; left−49/-1/38, right 53/0/25), superior cerebellum (SCb; left−25/-56/-21, right 16/-59/-21), thalamus (Th; left−10/-20/11, right 7/-20/11) and right postcentral gyrus (PCG; 37/-34/53) (Figure 1). According to the predefined coordinates in the study by Wang et al. (37), a 10 mm diameter sphere for each ROI was created, which guaranteed that there was no overlap between each pair of ROIs according to their Euclidean distance. By averaging the BOLD time series across all of the voxels within each ROI, we obtained the representative mean time series for each subject. Pearson correlation coefficients between the regional mean time series of all possible pairs of nodes were calculated to define the edges, thus resulting in a 21 × 21 correlation matrix for each subject. Due to the Interpretation unclear of the negative correlations (38, 39), our analysis of positive correlations was restricted.

### Network analysis

To further denoise spurious correlations between regions, we retained only the surviving correlations at the significance level *P* < 0.05 threshold (Bonferroni correction). A correlation threshold-defined by significance level has been used previously in brain network studies (40–43). At last, each correlation matrix was subjected to a threshold and converted into a binary matrix, wherein the entry ***a_ij_*** = 1 if the absolute value of the Pearson correlation coefficient between regions ***i*** and ***j*** was larger than the threshold and ***a_ij_*** = 0 otherwise. Additionally, each edge was weighted based on its functional connectivity.

### Network metrics

GRETNA software (http://www.nitrc.org/projects/gretna) was used to perform graph theory analysis of motion execution network (41), and calculate the global network and node level indicator. The formulation, usage and interpretation of these network measurements are reviewed in Rubinov et al. (44).

#### Small-world attributes

The most frequently used metrics are the small-world properties of brain networks, including clustering coefficient Cp, characteristic path length Lp, normalized clustering coefficient Gamma, normalized characteristic path length Lambda and small-worldness Sigma (45). CP measures the density of cliquishness or local density of the network, which characterizes network segregation. In network integration, *Lp* measures the extent of average connectivity or overall routing efficiency of the network. By scaling the average clustering coefficient and characteristic path length of 100 matched random brain networks, these random brain networks have the same number of nodes, edges and degree value distribution as the real brain networks (46), thus resulting in a normalized clustering coefficient Gamma and normalized characteristic path length Lambda. The two measurements can also be combined into a simple quantitative metric (small-worldness, Sigma = gamma/lambda).

#### Network efficiency

Small-world properties have been found in brain networks, enabling the efficient transfer of parallel information at relatively low costs (47). From the perspective of information transmission, network efficiency is a biologically relevant metric for describing brain networks (47, 48). The network efficiency at the global and local levels was calculated. The global efficiency *Eg* represents the capability of parallel information transfer over the network. The local efficiency Eloc reflects the fault tolerance of the network, which indicates how well the information is communicated within the neighbors of a given node when this node is eliminated.

#### Nodal degree

Node degree refers to the sum of the number of connections (binary graphs) or connection weights (weighted graphs) of all edges directly connected to a given node. Due to its simplicity and high test-retest reliability, node degree is the most commonly used index to measure node centrality (49, 50). An node with a high nodal degree is considered to be a hub, which is highly connected to other nodes.

### Statistical analysis

#### Differences in clinical variables

All of the statistical analyses were performed by using the SPSS 21.0 software package (SPSS, Chicago, Ill). The age, HAMA and HAMD scale scores of the WD and HC groups were compared by using the Wilcoxon rank-sum test of two independent samples for the numerical variable data. The sex differences between the groups were detected by using the Fisher's exact probability method. The mean framewise displacement (FD) of the two groups was calculated by using (http://www.nitrc.org/projects/gretna) (41), and a two-sample *T*-test was performed with this software.

#### Differences in network metrics

To determine whether global and nodal attributes were significantly different between WD and healthy controls, we performed a two-sample *T*-test for each brain network metric (small-world attribute, network efficiency, node degree), and the effects of age, sex, and mean FD were regressed. For global metrics, we adopted a significance level of *P* < 0.05. To address the issue of multiple comparisons, we tested the node degree at the expected significance level of 0.05 to see if it could be corrected for by FDR.

To detect connectivity measures (i.e., functional connectivity strength) of the motor execution network, we used a network-based statistical (NBS) approach (51) to locate specific connectivity that changed significantly in WD patients. First, a primary cluster definition threshold (*P* < 0.01) was used to identify a set of suprathreshold connections containing any connection components. Subsequently, adjusted *P*-values for each component were calculated using a non-parametric permutation test (5,000 permutations), and the significance threshold was set at *P* < 0.05. Likewise, age, sex, and mean FD were regressed out as covariates.

#### Relationships between network measures and clinical variables

Spearman's correlation analyses between network indicators and clinical variables were conducted for the indicators with significant group differences (i.e., onset age and duration of illness) within the WD patients. A significance value of 0.05 was used for multiple comparisons in correlation analyses.

## Results

### Demographic and clinical characteristics

Demographic and clinical data are presented in Table 1. Briefly, the two groups did not differ in age (Wilcoxon rank-sum test, ***Z*** = −1.631, *P* = 0.103) or gender (Fisher's exact probability method, *P* = 1.000). The mean FD also did not differ significantly between WD patients and healthy controls (Wilcoxon rank-sum test, ***Z*** = −0.923, *P* = 0.356). Anxiety and depression were used as additional exclusion criteria for participants, and there was no significant difference between HAMA and HAMD.

**Table 1:** Demographic and clinical characteristics.

**Characteristics**	**Wilson's disease patients**	**Healthy controls**	**Statistics**	***P*-value**
Number of subjects	23	15		
Age (years)	22.30 ± 5.49	24.67 ± 5.21	*Z* = −1.631	0.103^a^
Gender (female/male)	11/12	7/8		1.000^b^
meanFD	0.093 ± 0.043	0.104 ± 0.048	*Z* = −0.923	0.356^a^
Onset age (years)	13.48 ± 4.57	-		
Duration of illness (years)	8.83 ± 5.10	-		
UWDRS-IB	6.87 ± 2.34	-		
HAMA	1.96 ± 0.93	1.86 ± 0.99	*Z* = 0.381	0.703^a^
HAMD	2.43 ± 1.20	2.13 ± 1.06	*Z* = 0.742	0.458^a^

The data are shown as the mean ± SD. WD, Wilson's disease patients; HC, healthy controls; FD, frame-wise displacement; UWDRS-IB, Uniform Wilson's disease rating scale part I, B content; HAMA, hamilton rating scale for anxiety; HAMD, hamilton rating scale for depression.

a The *P*-value was obtained by Wilcoxon rank sum test.

a The *P-*value was obtained by Fisher's exact probability method.

### Global topological organization of the motor execution network

**Figure 2:**
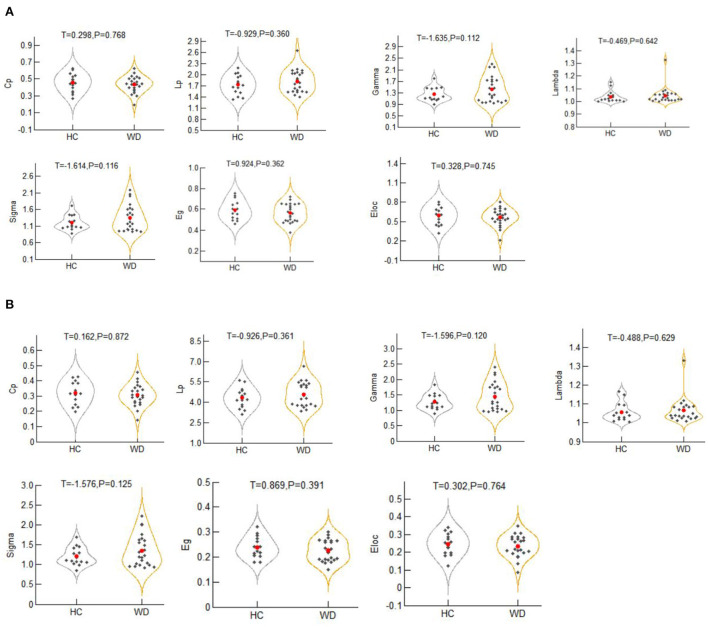
The global metrics of the binary graphs **(A)** and weighted graphs **(B)** of the motor execution network in Wilson's disease patients and healthy controls. Error bars represent standard errors. Cp, clustering coefficient; Lp, characteristic path length; Gamma, normalized clustering coefficient; Lambda, normalized characteristic path length; Sigma, small-worldness; Eloc, local efficiency; Eg, global efficiency; HC, healthy controls; WD, Wilson's disease patients.

The global indicators of the binary graphs of the motor execution network in WD patients and healthy controls are shown in Figure 2A. Compared with the random network, the motion execution networks of the WD and healthy controls have high agglomeration coefficients (i.e., Gamma >1), but almost the same characteristic path lengths (i.e., Lambda ≈1), indicating that both groups exhibit typical small-world topological properties. Nevertheless, there were no significant intergroup differences in *Cp, Lp*, gamma, lambda, Sigma, *Eg* or Eloc (*P* > 0.05).

The global indices of the weighted graphs of the two groups of motion execution networks are shown in Figure 2B. Compared with the random network, the motor execution networks of the WD and healthy controls have high agglomeration coefficients (i.e., Gamma >1), but almost the same characteristic path lengths (i.e., Lambda ≈1), indicating that both groups have typical small-world topological properties. No significant differences in *Cp, Lp*, gamma, lambda, sigma, *Eg* or Eloc were identified between the two groups (*P* > 0.05).

### Nodal topological organization of the motor execution network

**Figure 3:**
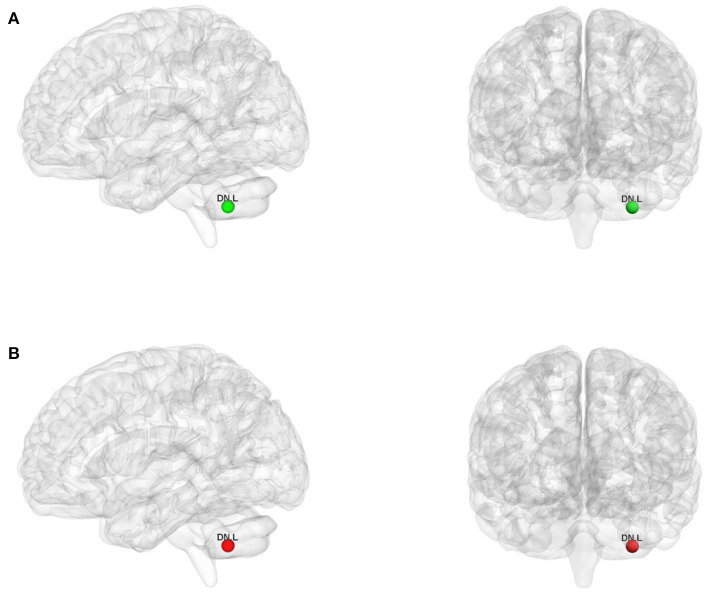
The nodes with increased nodal degree in Wilson's disease patients for the binary graphs **(A)** and weighted graphs **(B)** of the motor execution network. Node sizes represent *T* values. DN, dentate nucleus; L, left; R, right.

For the binary graphs, the WD patients exhibited an increased nodal degree in the left DN (*T* = −2.284, *P* = 0.029) compared to the healthy controls (*P* < 0.05, FDR corrected, Figure 3A). For the weighted graphs, the WD patients also exhibited an increased nodal degree in the left DN (*T* = −2.101, *P* = 0.043) compared to the healthy controls (*P* < 0.05, FDR corrected, Figure 3B).

### Disrupted functional connectivity strength in WD patients

**Figure 4:**
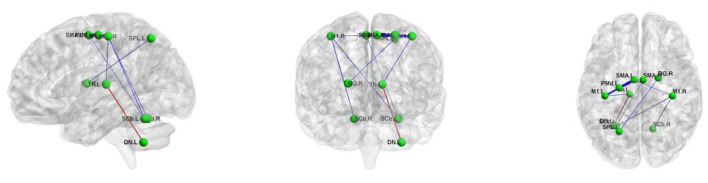
The connected component with altered functional connectivity strength in Wilson's disease patients. Edge colors represent increased (red) and decreased (blue) functional connectivity strength in the patients. Edge sizes represent *T* values. BG, basal ganglia; DN, dentate nucleus; L, left; M1, primary motor cortex; PMd, dorsolateral premotor cortex; R, right; SCb, superior cerebellum; SMA, supplementary motor area; SPL, superior parietal lobule; Th, thalamus.

Using the non-parametric NBS method, we found that individual connectivity components showed changes in functional connectivity strength in WD patients (*P* = 0.01, corrected, Figure 4). The component consisted of 10 edges and 11 nodes. Among them, the functional connectivity between left DN and left Th (*T* = −2.428, *P* = 0.024) increased in WD patients. In addition, WD patients had decreased functional connectivity strength in connections between the left SPL and right BG (*T* = 2.334, *P* = 0.030), left SMA and left M1 (*T* = 2.749, *P* = 0.012), left Th and left M1 (*T* = 2.312, *P* = 0.031), left SCb and right M1 (*T* = 2.344, *P* = 0.029), right SCb and right M1 (*T* = 2.345, *P* = 0.029), left SMA and right M1 (*T* = 2.299, *P* = 0.032), left SMA and left PMd (*T* = 3.004, *P* = 0.007), right SMA and left PMd (*T* = 2.240, *P* = 0.036) and right SMA and left SCb (*T* = 2.278, *P* = 0.033).

### Correlation analyses

There was no correlation between the network metrics and the clinical variables in the WD patients (i.e., onset age and duration of illness) were found.

## Discussion

Based on resting-state fMRI data and graph theoretical approaches, this study investigated the topological organization of the motor execution network in WD. Our main findings were as follows: (1) at the global level, there were no significant intergroup differences in the global topological organization of the motor execution network between the two groups; (2) at the node level, the nodal degree was higher in WD patients in the cerebellum; and (3) at the edge level, a cerebello-thalamo-striato-cortical circuit with altered functional connectivity strength in WD patients was observed. Specifically, the functional connectivity strength between the cerebellum and thalamus was increased, which may be a compensatory mechanism for WD dyskinesia, whereas the cortico-thalamic, cortico-striatal and cortico-cerebellar connections showed decreased functional connectivity strength, thus suggesting possible damage between the various levels of the nuclei that control movement. According to these findings, motor execution network topology may be disrupted in patients with WD, which may lead to motor dysfunction in these patients.

Based on graph theory, the human brain is considered a small-world network with high capabilities for localization and global integration (19, 52). In the current study, *via* the small-world model and efficiency measures, WD patients as well as healthy controls executed motor commands efficiently using small-world topologies. As far as small-world and efficiency metrics were concerned, the two groups did not differ significantly. A shift of this nature has been observed in several graph-theoretical studies of other neuropsychiatric diseases, including pediatric posttraumatic stress disorder (53, 54), attention-deficit/hyperactivity disorder (55), concussion (56), schizophrenia (43) and temporal lobe epilepsy (57). These studies are inconsistent with our findings. Different imaging modalities (fMRI *vs*. EEG), states (rest *vs*. task), and network scales (whole-brain network *vs*. motor execution network) may explain the inconsistent results. In addition, to ensure that the MRI procedure could be performed and to reduce the artifact generated by head movements as much as possible, we selected WD patients with less obvious head movements.

In addition to the global topologies, the motor execution network nodal attributes were further studied. Within the motor execution network, nodes play a central role in information transport and integration (58), and a greater number of nodes were found in the cerebellum of WD patients. Previous MRI studies of WD have found that abnormal striatum on magnetic resonance images is associated with parkinsonian symptoms, abnormal dentate thalamic tract is associated with cerebellar symptoms, and abnormal pontine cerebellar tract is associated with parkinsonian symptoms (59). A study combining VBM and ROI analyses found that the volumes of the caudate nucleus, putamen, globus pallidus, thalamus, brainstem and cerebellum of WD patients with the nervous system were lower than those of patients with hepatic presentations (60). This change in brain volume could be seen as evidence of brain shrinkage, but unfortunately, they didn't do a longitudinal study. However, Smolinski et al.'s long-term longitudinal study on WD patients (3) made up for the deficiency in this aspect. They found that the incidence of cerebral atrophy in WD patients with neurological symptoms was significantly higher than that in patients without neurological symptoms, and the rate of cerebral atrophy was related to the degree of neurological function damage in patients. Moreover, a study using advanced techniques of diffusion tensor imaging (DTI) to assess damage to the cerebellar-thalamo-cortical network found that lesions in the basal ganglia, thalamus, and cerebellum may compromise the basal ganglia-thalamic-cortical circuit or the dentate nucleus-red. Disruption of nucleo-thalamic (DRT) tract connections and eventual disruption of the cerebellar-thalamo-cortical network, which may cause clinical extrapyramidal symptoms (61). Given these previous findings, it is easy to speculate that the increased cerebellar nodal degree of the motor executive network may be a compensatory effect to overcome WD-related structural damage, which may also be a compensatory effect on motor dysfunction in WD. It cannot be ruled out, however, that WD-related motor deficits may result from a more highly connected cerebellum.

In terms of edge abnormalities, we found altered cerebellar-thalamo-striatal-cortical circuit functional connectivity strengths in WD patients. Specifically, the functional connection strength between the left DN and left Th was increased in WD patients, thus indicating that the functional connection strength between the cerebellum and thalamus was increased. The cerebellum maintains the balance of the body and the synergistic movement of the limbs. For example, the ancient cerebellum is responsible for maintaining body balance and coordinating eye movements; the old cerebellum is responsible for controlling muscle tension and coordinating muscle movements; the new cerebellum is responsible for controlling the planning and coordination of fine motor movements of the limbs; the thalamus anterior ventral nucleus and ventral lateral nucleus mainly receive afferent fibers from the dentate nucleus of the cerebellum, globus pallidus and substantia nigra; and after relaying from these areas, fibers are projected to the somatomotor center to regulate somatic movement. The increased functional connection strength between the two areas may be a compensatory mechanism for WD dyskinesia. In WD patients, the left SPL and right BG, left SMA and left M1, left Th and left M1, left SCb and right M1, right SCb and right M1, left SMA and right M1, left SMA and left PMd, right SMA and left PMd and right SMA and left SCb exhibited decreased functional connectivity strengths, thus indicating that cortico-thalamic, cortico-striatal and cortical-cerebellar connections showed decreased outgoing functional connection strength. Due to the disorder of copper ion metabolism in WD patients, excessive copper is deposited in the extrapyramidal system and cortex, which damages the corresponding nuclei. The striatum is an important part of an extrapyramidal system and a key part of motion control. When the striatum is injured, it can produce two different symptoms: increased muscle tone associated with too little movement, and decreased muscle tone associated with too much movement. The cerebral cortex is the highest level of the central nervous system and the highest level of control of bodily movement. The functional connectivity between the cortex-thalamus, cortex-striatum and cortex-cerebellum was reduced in this study, thus indicating that there may be damage between the various levels of nuclei that control movement. Previous resting-state fMRI studies have revealed decreased or increased functional connectivity of various networks in WD. For example, Han et al. reported that compared with healthy people, the default, attention and functional connectivity strength of the basal ganglia network were reduced in patients with WD (62). Moreover, Jing et al. showed that WD patients altered large-scale functional brain networks compared with healthy controls (63). Another study found that the decrease in functional connectivity strength in WD patients occurred primarily in the basal ganglia and thalamus, whereas the increase in functional connectivity strength occurred primarily in the prefrontal cortex (64). In contrast, our study revealed the coexistence of increased and decreased functional connectivity in the motor executive network, not only supporting previous findings but also providing unique insights into the neuropathology of WD.

There were still some limitations to this study. First, we did not assess the motor function of the participants. Due to the lack of relevant information, we were unable to further investigate its relationship with network indicators. Second, we only focused on the internal network connectivity of the motor network, but the internetwork connectivity between the motor network and other networks (such as the default mode network, salience network and executive control network) may also be associated with WD-related motor impairment, so it is worthy of further investigation. Third, to ensure that WD patients can undergo magnetic resonance examinations and to minimize the artifacts caused by head movements as much as possible, we selected patients with decreased head shaking, which may have an impact on our results. Lastly, due to the relatively small sample size, our findings are preliminary and need to be confirmed. As a result, future studies should include a larger sample size.

Of course, this study still has some limitations. First, the sample size is relatively small. Wilson's disease is a rare disease. It is difficult for patients to focus on systematic scale testing for a long time. Even if patients are asked to keep their heads still during MRI scanning, some patients still have difficulty in doing so. In the future, we will recruit more subjects to improve the data and use a larger sample size to verify the validity of these findings. Second, we only focused on the internal network connectivity of the motor network, while the internetwork connectivity between the motor network and other networks (such as the default mode network, salience network and attention network) may also be associated with WD-related motor impairment, so it is worthy of further investigation. Third, to ensure that WD patients can undergo magnetic resonance examinations and to minimize the artifacts caused by head movements as much as possible, we selected patients with decreased head shaking, which may have an impact on our results. Finally, the functional areas of the brain are generally irregular. We defined the nodes of the motion execution network by drawing balls (coordinates as the center, creating spheres with a certain radius), which may not necessarily include all the functional brain areas. This may have a certain impact on our results. In the future, we will extract various brain regions of the motor execution network by anatomical segmentation (65, 66) to verify our results.

Overall, we used resting-state fMRI to study WD motor execution network configurations. We found that WD patients have topologically dysfunctional motor executive networks, increased node degrees in the cerebellum and altered functional connectivity strengths of the cerebellum-thalamo-striatal-cortical circuit. It may be possible to develop biomarkers for early diagnosis of WD, therapeutic targets and biomarkers to help predict the pathophysiology of motor impairments in WD from these findings.

## Data availability statement

The raw data supporting the conclusions of this article will be made available by the authors, without undue reservation.

## Ethics statement

The studies involving human participants were reviewed and approved by Medical Research Ethics Committee of First Affiliated Hospital of Anhui University of Chinese Medicine. Written informed consent to participate in this study was provided by the participants' legal guardian/next of kin. Written informed consent was obtained from the individual(s), and minor(s)' legal guardian/next of kin, for the publication of any potentially identifiable images or data included in this article.

## Author contributions

LZ, HY, and YW participated in the conception and design of the trial. LZ, HY, QShe, ZL, HZ, YX, YC, JW, ZY, MW, and JH acquired the data. HY collated the data and LZ analyzed the data. LZ and HY participated in drafting the manuscript. YW, TD, WY, LX, ZH, and QShi provided help and guidance. All authors contributed to the article and approved the submitted version.

## Funding

The work was supported by the Natural Science Foundation of Anhui Province (No. 1508085MH199); the Research and Training Project for the Fourth Batch of National Top Talents of Traditional Chinese Medicine (Clinical and Basic) [Documents from National Administration of Traditional Chinese Medicine (2017) No. 24]; Xin'an Key Laboratory of Medical Science, Ministry of Education (No. 2020XAYX09); Graduate Science and Technology Innovation Fund of Anhui University of Traditional Chinese Medicine (No. 2020ZD02).

## Conflict of interest

The authors declare that the research was conducted in the absence of any commercial or financial relationships that could be construed as a potential conflict of interest.

## Publisher's note

All claims expressed in this article are solely those of the authors and do not necessarily represent those of their affiliated organizations, or those of the publisher, the editors and the reviewers. Any product that may be evaluated in this article, or claim that may be made by its manufacturer, is not guaranteed or endorsed by the publisher.
